# A qualitative study of CVD management and dietary changes: problems of ‘too much’ and ‘contradictory’ information

**DOI:** 10.1186/1471-2296-15-25

**Published:** 2014-02-04

**Authors:** Samantha B Meyer, John Coveney, Paul R Ward

**Affiliations:** 1 University of Waterloo, School of Health and Health System, 200 University Ave West, Waterloo, Ontario N2L 3G1, Canada; 2 Discipline of Public Health, Flinders University, Adelaide 5001, Australia

**Keywords:** General practice, Cardiovascular disease, Lifestyle, Diet, Australia

## Abstract

**Background:**

Nutrition education for cardiovascular disease (CVD) management is not effective for all population groups. There is little understanding of the factors that hinder patients from adhering to dietary recommendations.

**Methods:**

37 interviews were conducted with people living with CVD in Adelaide, Australia. Recruitment occurred via General Practitioner (GP) clinics and hospital cardiac rehabilitation programs. Participants were either receiving preventive treatment or active treatment for established CVD.

**Results:**

The volume and contradictory nature of dietary information were the most prominent barriers to making changes identified in interviews, especially by order participants.

**Conclusion:**

Patients will seek out, or come into contact with information which contradicts advice from their GPs. The volume of information may lead them to resort to old and familiar habits. GPs play a valuable role in highlighting key take-home messages and reliable external sources of information. The findings have implications for GP practice given that lifestyle changes are a cost- and clinically-effective means of managing CVD.

## Background

Globally, cardiovascular diseases (CVDs) are the biggest cause of death worldwide with more than 17 million deaths occurring in 2008 [1]. Within Australia, cardiovascular diseases are the leading causes of mortality and morbidity [2], and when last calculated, were responsible for 21.9% of total disability-adjusted life years (DALY) in 1996 [3]. Healthy eating has been identified as a key strategy for the self-management of chronic illnesses such as CVD [4,5]. As such, the promotion of healthy eating has been recognised as an integral part of high-quality primary care [6].

General Practitioners (GPs) play a central role in the on-going management of patients with CVD and are a valuable source of information regarding how to self-manage. However, it is widespread in the literature that patients have difficulty coping with strategies and mechanisms as a means of changing their diet [7,8]. It is evident that nutrition education based exclusively or predominantly on providing knowledge and skills has not been shown to be effective [9]. This has direct implications for GPs given that lifestyle is a cost- and clinically-effective means of managing CVD. Although there have been studies and reviews of patients’ self-management of CVD [5,10,11], further research is required to address the barriers to self-management in individuals with heart disease in order to provide an appropriate guide for intervention strategies [12].

## Methods

Individuals from 33 different postcodes across Adelaide, Australia were interviewed regarding barriers to following the dietary recommendations of the healthcare professionals treating their CVD. The interview guide consisted of questions regarding the difficulties in making changes to diet following diagnosis with CVD. Interviews were conducted in the participants’ homes or at locations of their choice (two at a shopping mall, one at a café, one over a picnic).

Participants were recruited via South Australian cardiac rehabilitation programs and South Australian General Practitioner (GP) surgeries. One hundred patients were contacted by their GP regarding the research and the primary author attended four cardiac rehabilitation centres (roughly 20 participants in each) to recruit participants. No remuneration was offered for participation in the study.

Participants were chosen to ensure diversity with regards to their diagnostics and interventions for CVD, socio-economic status (SES), sex and age. The final participant group consisted of 22 males and 15 females (due to a higher prevalence of men in the general population with CVD [13]), 18 undergoing preventative treatment for CVD (taking a statin to reduce cholesterol), 19 with acute CVD (have had heart surgery or a cardiac event) and ages ranging from 32–80. Participants ranged from being newly diagnosed with CVD or having lived with CVD for over ten years. Some participants had attended cardiac rehabilitation programs following their heart surgery/heart attack, while others had not accessed any form of primary prevention other than being prescribed medication. Participants were from a range of SES (based on income, education, area of residence) though with a slight overall tendency towards more middle-class brackets. Data were collected between October 2008 and September 2009.

Interviews were audio recorded and transcribed. Thematic analysis [14] of the data presented in this paper was carried out via the coding of reoccurring themes using Nvivo [15]. Data collection continued until saturation of themes was reached. A coding structure was developed with the aim of exploring and understanding the factors that make it hard for individuals with CVD to adhere to the guidelines provided to them regarding diet. Given the differences across participant demographics, the analysis was informed by considerations of the data within the wider context of each respondent. The final stage was to group codes into themes according to difficulties identified by participants.

Research ethics approval was obtained from Flinders University Social and Behavioural Research Ethics Committee (Approval number 4245) prior to commencement of study. All respondents provided written consent prior to data collection.

## Results and discussion

Participants said that they are confronted with too many sources of diet-related information, which limits their capacities to make decisions about what foods to purchase and consume. Specifically, participants reported that the volume of information they were confronted with and the contradictory nature of information they received both within and external to medical practice, made it difficult to adhere to recommendations.

Many participants stopped following recommendations, from both healthcare professionals and external sources (the Internet, television etc.) because they found it difficult to know what information was accurate. Participants talked about being “bombarded” with information. As a result, participants suggested that they rely on ‘common sense’ rather than the dietary information they had been given.

“I think it’s just, it’s just too much. There’s too much information. It’s too difficult to, to sift so that I don’t do it.” (Interviewee 36)

“Well, I’ve, it was a sort of, you know, sat in the doctor’s surgery and he said ‘well you ought to try cutting out fats and…’. I might, he might have given me a couple of… but ah, I mean now you’re bombarded …” (Interviewee 34)

The above quotes were given by men ages 62 and 65. The difficulty with the volume of information was more prevalent in male respondents, especially those who were older. However, it was also noted in female participants. For example, interviewee 16 referred to society as being “overknowledged”, referring to the fact that we have access to so much information that making choices become too difficult.

Similarly, older participants reported that they were given advice to follow food labels as a way to eat healthy but did not know how to read them, or what to look for.

“Oh yeah. Too many numbers on them [food labels] still, you know, and you don’t know what they are.” (Interviewee 20)

“I stopped putting my glasses on [when grocery shopping] because I thought well if I can’t buy anything, I’ll just try to be sensible.” (Interviewee 11)

They identified that the information given about labels was too complex and difficult to follow. The latter quote was provided by a man who found that grocery shopping was difficult because of all of the label reading. As a result, he chose to stop reading labels all together. Many older participants were also sceptical of marketing ploys used to sell seemingly healthier products. This further complicated their choices about what they should be purchasing. For example, Interviewee 24 said: “You read these things [labels] when you go shopping – how much fat and all that but you don’t know what to believe half the time.”

In addition to too much information, participants across all demographic groups noted that the information they received about healthy eating was conflicting and contradictory, thus increasing the complexity of their decisions about what advice to follow:

“… I’d read all this information before from the Heart Foundation, they, they didn’t exactly gel together [with information he received at a dietitian’s session at cardiac rehabilitation].” (Interviewee 37)

Many participants also noted there to be certain foods, for example eggs, that are inconsistently ‘good’ or ‘bad’ to eat:

“Yes in some cases but it, it, every year it seems there is new studies so it brings out something else that you are supposed to follow, or not follow or change. So, it changes all the time.” (Interviewee 7)

“I was told that you shouldn’t eat too many eggs. Since then I’ve heard studies that said ah but you can have as many eggs as you want but I am still keeping it low because, well until they have absolutely confirmed I’d rather not.” (Interviewee 8)

Interviewee 8 later in the interview went on to discuss this in greater detail:

“But, she [a dietitian] said “ah, no, no, no. That’s out of date. It’s alright to have all these eggs”. Since then I have read in the paper that, you know, they’ve done further studies and they’ve said that eggs aren’t necessarily a bad think but I’m still – I’d rather ere on caution than worry – you know, that till it’s absolutely proven.” (Interviewee 8)

“I mean, at, when I, in 87 [1987] – I was told for instance, absolutely no avocado you know… but they’re good fats…So it’s changed.” (Interviewee 12)

Recent literature has identified a number of factors that act as barriers to dietary changes in patients with heart disease. These include: a lack of professional support, temptations or difficulty with self-discipline, food preference, insufficient knowledge, unhelpful social contacts (e.g. household members), personal problems (e.g. disruption of daily routines), the perception that lifestyle changes do not lead to improved health, and difficulties in managing added dietary requirements for comorbidities [12,16-18]. However, novel in this research are the two most central barriers identified by interviewees: the volume and contradictory nature of information.

The barrier of ‘too much’ information has been identified with other chronic conditions. For example, Briggs et al. found that when patients consulted more than one medical professional, it lead to large quantities of information that was difficult for patients to digest. The finding that information from healthcare professionals and external sources (e.g. the Internet) is contradictorys (e.g. eggs) has also been noted elsewhere [19]. This finding is consistent with research conducted by Ward et al. whereby participants said the confusing or conflicting information they received led them to ignore health messages about food as a means of reducing and managing uncertainty. Confusing and often contradictory information regarding food and nutrition has been demonstrated to lead to a sense of paralysis or stasis when making food choices [20,21]. Therefore, when faced with diet-related diseases, an individual may adhere to existing and familiar food habits, rather than making the recommended changes.

## Conclusion

Despite the education programs and services offered via primary healthcare to assist individuals to eat healthy foods as a means of managing their CVD, the interviews identify barriers that hamper participants’ capacity to do so. Given the role of primary care providers, namely GPs, in the management of CVD, we suggest that GPs ensure that patients are being appropriately informed about scientifically-based information [7]. We also recommend that GPs be cognisant of the need to present information in a manner that patients can understand, utilise and digest*.* This may mean that quality over volume needs to be considered and that healthcare professionals might consider pointing patients to valuable sources of external information if appropriate. The need for appropriate guidance is especially important for older patients.

### Limitations of this study

We acknowledge that the results of this research are not generalizable as the participants consist of a small non-representative sample. Additionally, we acknowledge that there are additional factors (e.g. taste, affordability, convenience) that will ultimately affect consumption patterns. Nevertheless, given the relative paucity of data in this area of interest, the current results add substantially to our understanding of the barriers to adhering to the recommendation of healthcare professionals.

## Abbreviations

CVD: Cardiovascular disease; GP: General practice.

## Competing interests

The authors declare that they have no competing interests.

## Authors’ contributions

SBM was involved in the research design and analysis, and conducted all interviews. PRW and JC were involved in the research design and the analysis of the data. All authors contributed to the drafting of the manuscript. All authors approved the final manuscript.

## Pre-publication history

The pre-publication history for this paper can be accessed here:

http://www.biomedcentral.com/1471-2296/15/25/prepub
